# Digital treatments for affective disorders: an integrated overview

**DOI:** 10.1192/j.eurpsy.2024.76

**Published:** 2024-08-27

**Authors:** U. Volpe

**Affiliations:** Department of Clinical Neurosciences/DIMSC, Università Politecnica delle Marche, Ancona, Italy

## Abstract

**Abstract:**

Affective disorders represent a category of psychiatric syndromes with high prevalence and associated disability. While effective, both pharmacological and psychosocial, treatments are available for depression and bipolar disorder, the many therapeutic needs of affected patients are far from being properly addressed under routine conditions. Along the past decade, several digital treatments, tools and approaches have been developed and tested in clinical settings, showing an highly promising potential to fill the treatment gap of affective psychopathology. In more detail, reviewed here will be telepsychiatry solutions for affective disorders, also encompassing the available officially approved digital therapies for major depression and bipolar disorder. Furthermore, the impact of artificial intelligence, serious gaming, social media and virtual/augmented reality in the treatment of mood disorders will be also discussed, in the light of the most recent research evidence on these topics.

**Disclosure of Interest:**

None Declared

